# Abstracts and Gleanings

**Published:** 1887-10

**Authors:** 


### ABSTRACTS AND GLEANINGS.

**Proper Time to Give Medicines.**—Prof. G. See makes some practical remarks that are quite different in some respects from the teachings of the books, and also from custom; but, as they are the results of his nearly forty years' experience in hospitals, they can be followed with certainty. "When is the exact moment to give drugs so that the system will best accept them? There are a few that may be given any time you like, but these are the exception:"

*Cod-Liver Oil.*—"What causes absorption of this oil? The action of the pancreatic and hepatic secretions. Given fasting, it will most likely cause vomiting, as the juices are not present; for secretion only commences when there is something in the stomach. Children take it well, and the reason is that in them the sense of taste is imperfect. It must be given, then, so that it will pass quickly on to where it can meet the pancreatic juice: so give it at meals, just after taking soup; and it can also, curious to say, be well digested without any 'returns,' if taken the last thing at night on going to bed. Cod-liver oil contains fatty acids, more so than any other oil, and absorption proceeds better with it, as an emulsion is not so much needed as in other oils."

*Emetics.*—"When the intention is to have only mucus vomited, give these fasting; but in digestion, etc., exhibit after eating, so that there will be something to vomit."

*Purgatives.*—"Here there must be a division. Carlsbad, Hunyadi Janos, and such like purgative waters should be given at once on rising; and always in hot water, to precipitate the elements; if given cold they are often vomited. Magnesia salts, on the other hand, require time, and should be taken at night. Next we come to purgatives that must never be given fasting: these are the drastics, such as jalap, aloes, etc." (Here Dr. See tells a funny story of his young days, when patients were few and far between, and he got one to whom he ordered a compound aloes pill, with other things, and ordered it to be taken before each meal, with a result that the unfortunate patient had a vomiting-fit each time, and sent at once for the doctor's bill and requested him not to call any more." "These, then, should be given in the middle of a meal;" don't say during, for, like before a meal, many people want to know the exact moment, and don't understand if you mean an hour before meals or at meals: so be very precise."

*Mineral Waters.*—Dr. See condemns the usual custom of putting these into the wine which is drunk at table, and he says they spoil both wine and the digestion. He calls attention to the fact, that at Vichy, and all the mineral water stations, the water is always given fasting and sometime before a meal. The object being to increase the secretion of the gastric juice, they must be given before meals—and not just before, but at least half an hour before. Vichy, administered in this way, gives better results, than when it is used to turn red wine into a sort of ink.

2

Bitters, cinchona, wines, etc., are what are called tonics, on account of the tannin that exist in them; these and acids should be taken just at desert, when the meal is over; certainly never before meals.

*Iron.*—It will precipitate the gastric juice taken before meals, therefore take it when there is something in the stomach to prevent this. It is not known how it gets into the circulation, because it is not seen to go out. In any case, give it with meals.

*Pepsine.*—In supposing that there is some virtue in pepsine, which has not been proved, it should be given just at the end of a meal to assist the digestion of it.—*Technics.*

**Hygiene of the Hair.**—Dr. Foley (*New York Medical Record*) says: The end we seek in building up a scanty hair crop is a proper amount of blood-supply, through friction and hair-tonic:

℞. Acid. carbolic.................................ʒ ss,

Tr. nucis vom.....................................ʒ ij,

Tr. cinchonæ rubr.................................℥ j,

Tr. cantharidis...................................℥ ss,

Aq. cologniensis..............................

Ol. cocois.......................... ...aa q. s. ad ℥ iv. M.

Apply once or twice a day to the scalp by means of a soft sponge. This will prevent the hair from falling out if it does not produce a luxuriant crop.

Fine-toothed combs should be avoided, and used only from a sportsman's point of view—"to catch game." They have a tendency to peel off the scarf skin and leave a denuded surface below, which is apt to end in disease, pityriasis, etc. Dr. Leonard gives the following trite remarks in selecting a brush or comb:

"A hair-brush or comb with silvery bristles or teeth too sharp is not good; the scalp will be scratched by the one and the hair broken by the other. A proper brush is made up of bristles varying with the individual as regards the stiffness of them. The clusters should be evenly set into the back, equidistant from each other, so that the whole surface of the scalp to which it is applied will be touched by some one of the bristles-bunches. Then the clusters should be made up of bristles of slightly unequal length, so as to still further favor the brush in covering every part of the scalp; by this means every hair will be rubbed down on all sides, and there will be no streaks or spots of the scalp left untouched.

"A proper comb in one whose teeth are even and regular, with points not sharp but rounded. It should be held up to the light so as to detect any splitting or roughening of the teeth on the sides; for, if they are so roughened, injury to the hair through breakage of the shaft will result. Should the teeth through any cause become split, as you value your hair, the offending members should be carefully cut from the comb; the slight space on the scalp that would thus remain untouched would be of no moment. Wire brushes are nothing more than combs. They act as a stimulant to the scalp, but are not equal to good bristle-brush."

A good supply of oxygen is necessary for the healthy growth of hair; the head should be well aired. The hat has made sad havoc with many a caput. Endeavor to go bare headed as often as possible. When walking, lift the hat from off the head frequently, and, if the sun is not too strong, hold the hat in your hand awhile. The blue-coat school-boys formerly of Christ Church, London, who wear the costume of Edward VI, go bareheaded the year round. They wear no hats in the coldest days of winter. They are remarkably healthy, and have a redundant crop of hair, which lasts them a life-time. If we must wear a hat, let it be light in texture and well ventilated from the top. One reason that women keep their hair longer than men is that their head-gear allows of better ventilation. Business men sometimes wear their hats in their office, or have a special hat which they put on. This is very injurious. The brokers of Wall Street are noted for wearing their hats indoors as well as out-doors. They are also notorious for having bald heads. This may account for it. When the head is well shorn of its locks this does not apply.

**Pasteur and his New Communication**.—Since Pasteur first commenced his investigations into bacteriology, and after his discovery of the protection which he could give sheep, by inoculating them with an attenuated virus of charbon, the eyes of the scientific world have been turned toward that unpretending laboratory in Paris for further important revelations.

When he gave out to the world the fact that he believed that hydrophobia could be prevented by giving hypodermic injections of an attenuated virus of rabies, the knowing ones simply winked their eyes and said, "I told you so." It seemed that this great mind must evolve some important principle which would be as of great benefit to humanity, as inoculating the sheep had been to the flocks of France. While Pasteur has many violent enemies and opponents, still the bulk of the scientific world gave respectful attention to his words and simply said, "Prove what you say to be true and we will worship you as a benefactor of mankind." The question now is: Has he proven his theory? We think, from the present standpoint, we are compelled to admit, that Pasteur's method will, in a goodly proportion of cases, cause immunity from the disease; still we are well assured from M. Pasteur's own words, that in some instances it has failed to do that which he thought would be done, viz., protect. After reading the communication which he made before a recent meeting of the Academy of Sciences of France, we are compelled to think that Pasteur has himself come to the conclusion, that there is a flaw in his previous methods, as in the paper above alluded to he says: "That the method he has employed for the last two months was inefficient, and that he was now employing a new method," as he terms it, "consisting of repeating the treatment three times a day; in employing for the inoculations the spinal marrows, beginning with the oldest and ending with the most recent."

This seems strange, when we take into consideration the fact that on the 26th day of October, 1885, M. Pasteur affirmed he had discovered an infallible method for the cure of rabies after a bite.

It seems a matter of regret for all interested in this important subject, that the other observers working in this same direction and under different auspices, *i. e.*, different countries, have not been able to attain the same success as M. Pasteur.

We shall look with a good deal of interest to the next report, in which we shall expect to see some results recorded of the new departure.—*Medical Register.*

**Nitro-glycerine.**—Dr. Trussewitch, St. Petersburg, *Medical Woch.*, shows that the value of this drug in various affections, agina-pectoris migrane and neuralgia, which he describes as angio neuroses, as well as in sea sickness, anæmia, faintness, palpitation and other diseases depends upon the existence of an irregular distribution of blood, judging from a degree of pallor of the skin often co-existent with a weak pulse and small, rigid, radial artery. On the other hand, when headache and neuralgia occur in patients with chronic congestion of the subcutaneous veins of the face, nitro-glycerine is to be avoided, and it is of no value in asthma when the skin is reddened in consequence of emphysena. If, however, a pale face exists with angina pectoris, migrane, giddiness, shock, or seasickness, good results may be looked for from nitro-glycerine. The effect of the drug over the congestion of internal organs, is similar to that brought about by blood-letting, and in these congestions, whether of lung, brain, or kidney, when they are of temporary character, the pulse is generally found to be slow and of low tension. As a rule, the pulse is the best indication for the employment of nitro-glycerine, and is the most-trustworthy guide, as to the initial dose. The smaller the radical artery, the more rapidly it dilates under the action of the drug, and the less the secondary effects produced. The fuller the pulse, with a distended radial artery, with weak pulse, the greater the secondary, and less the general effects. Single drop does of the 1 per cent. solution are sufficient in case of small pulse, but with a full pulse the full effects cannot be produced with less than two-drop doses. With a soft artery and weak pulse sub-normal doses, only, should be given, a ¼ to ½ a drop. The burn ng sensations of pulsation, and pain in the head are the guides for increasing the dose. The best methods of administering nitro-glycerine, are to simply drop the solution on the tongue, or the use of tablets. Less satisfactory results are obtained when mixed with water.—*Technics.*

**Heroic Dose of Turpentine in Croup.**—In an obstinate and dangerous case of diphtheritic croup, which had extended into the larynx, after painting with boracic acid, and subsequently with a chloric acid application, without benefit, the child's condition becoming worse and worse, Dr. Lewentaner, of Constantinople, before resorting to tracheotomy, remembering a paper by Demlow, in which turpentine was recommended in these cases, determined to give it a trial, and so administered with his own hands a teaspoonful of the pure oleum terebinthæ, giving after it some warm milk. In a quarter of an hour the labored laryngeal breathing had given away to normal respiration sounds. That night the child slept well and was quite free from the brassy cough which had previously been present. The next morning he was quite lively and was found playing with his toys. All trace of the false membrane had disappeared from the pharynx, which merely presented a reddened surface. Convalescence was rapid and uninterrupted. The turpentine, however, caused an eruption on the face, trunk, and extremities, having much the same appearance as the rash of measles, but of a brighter red. The spots completely faded in two days, and was followed by no sign of desquamation.—*Lancet.*

**The Modern Treatment of Urethritis.**—Dr. George E. Brewer contributes an interesting paper on this subject to the *Journal of Cutaneous and Genito-Urinary Diseases*, May, 1887. The methods referred to are irrigation by bichloride of mercury of a strength of 1 to 10,000 to 1 to 60,000, and the retrojection of hot water:

"The apparatus employed in the method by hot retrojection consists in a tin pail, beneath which is fastened a platform for an alcohol lamp or Bunsen burner. The pail is connected by means of a long rubber tube, with a No. 18 flexible catheter. The whole is suspended from the ceiling by means of a cord and pulley, and can be raised or lowered at will.

"The patient, after having first passed his water, is seated on the edge of a chair over a large sized slop jar or pail. The catheter, well oiled, is passed into the urethra about five inches and the current of water started. This is at first about the temperature of the body, but gradually raised until it is as hot as the patient can bear. About two quarts are passed through at each sitting. These should be repeated at least twice a day.

"The result of this method of treatment with hot water alone, or combined with a small amount of tannin or sulphate of zinc, is to once allay the inflammatory symptoms, lessen and modify the character of the discharge.

"During the past six months the number of cases of urethritis treated at the out-patient department of Roosevelt Hospital was 212. Of these 139 were acute and 79 chronic. Of the acute cases 88 were classed as specific and 41 as non specific or doubtful. Of the chronic cases 44 were classed as chronic purulent urethritis, and 29 as gleet.

"Seventy-seven were treated by irrigation with bichloride of mercury, 46 by hot retrojection; the La Fayette mixture was employed in 11 cases, while 7 received no treatment, but were kept under observation."

Dr. Brewer offers the following conclusions:

"1. That in uncomplicated cases of acute gonorrhœal urethritis, treated by prolonged and frequent irrigation with bichloride of mercury, recovery may be expected within two weeks; that this period may be considerably shortened by the early inauguration of treatment, by inordinate physical exertion, and by indulgence in alcoholic and venereal excesses.

"2. That the retrojection of a hot solution of bichloride possesses all the advantages of the former procedure, and in addition causes a more rapid subsidence of the inflammatory symptoms, a greater feeling of comfort to the patient, and is attended with less annoyance and trouble.

"3. That in cases of acute non-specific urethritis, the favorable influence of each of these methods is strikingly apparent.

"4. That in cases of chronic purulent urethritis, no agent produces such rapid and permanent improvement as irrigation, especially when combined with astringents and heat.

"5. That the percentage of complications occurring in causes treated by these methods is far below that observed when the ordinary methods are employed."—*Southwestern Medical Gazette.*

**Abortive Treatment of Furuncles.**—Dr. Louis Heitzman, of New York, writes as follows: After it was shown, some years ago, that micro organisms are the actual causes of furuncles, it is rather surprising that parasiticides have not long ago been used in the local treatment of boils. It is well known that among the numerous parasiticides, salicylic acid often does excellent service in diseases of the skin, but in furuncles it has, to my knowledge, never been used. I therefore tried to abort furuncles with salicylic acid, plaster or salve, and the result was really surprising, for it acted beautifully. The first case in which it was used was that of a young man, who, for three weeks previously had suffered from a large number of boils of different sizes at the back of the neck. In spite of numerous incisions, new nodules appeared as soon as others were cured. The man suffered such pains that he was unable to sleep, and he could not turn his head in the slightest degree without most severe pains. When I saw him first, he had about one dozen larger and smaller elevated, indurated, inflammatory nodules on the left side of the back of the neck. I prescribed locally an eight per cent. salicylic-acid plaster:

℞. Acid. salicyl.................................ʒ ij,

Empl. saponat.................................℥ ij,

Empl. diachyl.................................℥ j.

When I again saw him, three days later, the pains had disappeared, he could move his head in every direction, and only a few superficial nodules remained; in three days more he was cured. I had opportunity to test the action of salicylic acid in furuncles several times, and almost every time with the best success. If applied early enough, it never took longer than six or eight days to cure furuncles of even a large size or long duration, and the relief to the patient was manifested in a very short time. It is immaterial whether the salicylic acid is used as a salve or in the form of a plaster, the effect is always the same, and a five or ten per cent. salicylic-acid salve is of the same service. I use the officinal Unguentum aquæ rosæ as the basis for salves, not only because it has an agreeable smell, but because it is harmless; and this cannot always be said of vaseline, which is sometimes not free from sulphuric acid. A pleasant way to use the salicylic acid is in the form of the ten per cent. mucill, which was the first introduced by Unna; it is used in the same way as the plaster, and causes a superfiscal formation of blisters, or a sloughing of the skin.—*Peoria Medical Monthly.*

**Sick Headache.**—Coffee, or its active ingredients, caffeine and guarana. often relieve lighter paroxysms. Chloral and croton chloral are of more or less service in most cases. Anstie believed that the administration of 20 grains of chloral, the patient at the same time keeping his feet in hot mustard water, and inhaling the steam from the mustard, was the ideal treatment for migraine. Bromide of potash affords relief in some cases, but it is usually necessary to give very large doses. A new remedy, antipyrin, has proved a valuable auxiliary in our treatment of migraine and other forms of headache. One or two doses of 10 or 15 grains, given at the beginning of an attack of sick headache, will often act like a charm in cutting it short. A still newer remedy, antifebrin, is said to act equally well.

In some very severe attacks hypodermics of morpine may be called for to procure relief, and even these may afford but very little benefit.

In our efforts to prevent the attacks of sick headaches, or lessen their frequency and severity, we should attempt to remove all the causes which have any influence in their production. In some instances stomach disorders, diseases of the womb or the like, either directly or indirectly, occasion their development. Wherever diseases of this character exist, they should, if possible, be removed.

Special remedies are sometimes used with the idea of preventing future attacks. Cannabis indica is a favorite with some physicians. Its use for a long time is said to have a very decided effect in some cases. I have, myself, very rarely resorted to any specific medication in these cases. When I did so it was to administer the bromides, and only at such times when the headaches appeared to occur with unusual frequency and severity. Periods of this kind, of longer or shorter duration, are not rare occurrences to those suffering with migraine. I have almost invariably found that 10 or 15 grains of bromide of potash, given three times a day at such times, would be productive of much benefit.—*Cincinnati Medical News.*

**Bicarbonate of Sodium in the Treatment of Gonorrhœa.**—The copious discharge which is so marked a feature in gonorrhœa is invariably acid in reaction, and this fact has even been suggested as a test for the nature of the disease. The specific bacillus, moreover, to which gonorrhœa owes its ready and peculiar infectiveness, can only flourish and multiply in an acid medium. Considerations of this kind have led Dr. Castellan, a French army surgeon, to try the effects of alkaline injections into the urethra in gonnorrhœa. The results obtained are reported by him to have been excellent. He begins by testing the reaction of the discharge, and then, if acid, injects a small quantity of a 1 per cent. solution of bicarbonate of sodium, the least irritating of the alkaline salts, repeating the injection several times daily. Under this treatment, the scalding accompanying micturition is at once alleviated, and in the course of a week a very marked diminution is brought about of the discharge. Convalescence was generally rapid, and the final results very satisfactory. The treatment is also applicable to gleet. The good results alleged to have been obtained naturally require to be confirmed by experience before we can call on our neighbor to rejoice. Reiterated disappointment has begotten scepticism, but the plan of treatment has the great merit of being inexpensive and absolutely free from risk. Even if the specific action of the alkaline solutions be not justified by observation, nothing but good could follow the employment of a weak solution of anything.—*Medical Press and Circular.*

**Treatment of Palpitation.**—During an attack of acute palpitation, medical treatment of a direct kind can only be palliative. It is a common practice to place the patient in the perfectly recumbent position, but as this position leads, frequently, to breathlessness and much discomfort, I never enforce it unduly. The sufferers usually find out the best position for themselves, and standing up, and even gentle walking backward and forward, commonly appear to bring relief, as if the general muscular action equalized the local overaction.

For the actual palpitation, digitalis is the only remedy I have found of any positive service, and it combines well with remedies which have a tendency to promote quickly the cutaneous and renal excretions. I usually prescribe the tincture of digitalis in 5 or 10 minim doses, with ½ a fluid drachm of nitric ether, and 2 fluid drachms of the liquor ammoniæ acetatis. In instances where there has been prolonged sleeplessness, with palpitation, I have combined morphia, in full doses, with digitalis, with good effect, adding the narcotic dose to the formula just named.

In general treatment I am accustomed to follow, whether the heart be organically sound or unsound, the same methods as those prescribed in my previous essay on intermittency. The organic bromides of iron, quinine, and morphia, and the mixture of iron carbonate, ammonia, and morphia (*Asclepiad*, Vol. I, p. 204), are excellent remedies. The only difference in treatment, in fact, relates to the use of alcohol, which, valuable in some cases of intermittency, is less compatible in cases of palpitation.

The rules already ordered for the management of cardiac apply equally to the epigastric palpitation. There is, however in cases of epigastric palpitation more frequent necessity to meet dyspeptic symptoms, including flatulency and consumption, by alternative and mild aperient correctives.—*Asclepiad.*

**Subnitrate of Bismuth as a Dressing.**—1. Subnitrate of bismuth possesses antiseptic properties at least equal to those of iodoform.

2. No poisonous effects are to be appreh nded as in the employment of iodoform.

3. The subnitrate of bismuth, being a chemically indifferent substance, does not irritate the wounds; secretion is diminished.

4. Its action is very prolonged, though not vigorous, so that the dressings do not require to be frequently changed, and rest is insured for the wounds.

5. There is no action at a distance, nor does any specific effect attach to it.

6. It does not afford protection against erysipelas and other wound diseases, at least no more than iodoform.

7. It is no disinfectant, but as an antiseptic it keeps the wounds pure.

8. All wounds capable of healing by first intention can do so when dressed with bismuth.

9. It also represents an excellent material for forming scabs, under which epidermis can grow over the wound. Its use on granulating wounds has not, however, been sufficiently studied as yet.—*Annals of Surgery.*

**Treatment of Dysentery.**—*Progress Medical.*—In a correspondence from Bombay, Dr. C. MacDowall, physician in the British army of East India, speaks with great enthusiasm of the treatment of dysentery by ipecacuanha. Like other friends of this treatment, such as Docker, Ewart, Cunningham, Malun, etc., he says that it is almost a specific, renders the disease easy to cure, and prevents the complication most feared, *i. e.*, hepatic suppuration. But he emphasizes, particularly, "that the remedy be given early in the disease, at the proper time and in the proper manner." The principles of the treatment are:

1. To give a large dose of ipecac, at least 30 grains, for an adult.

2. To prepare the stomach to accept and retain such a large dose by about 20 drops of laudanum an hour before giving the ipecac; also the application of a sinapism over the stomach; and to administer the ipecac in the form of large pills, not in solution. It must also be given at night, at the time of going to sleep, never in the morning, and not during the day, and no liquid is to be taken after the dose has been given.

Sometimes the patient vomits a little mucous towards the morning hours, but the greater portion of the remedy has by that time been absorbed. This treatment must be renewed every night, and usually the improvement is marked by the third morning, or sooner; blood, mucus, pain, all three having disappeared. A disease which formerly made us despiar has now lost its terror to us.

The opium may be substituted by a hypodermic injection of morphia. Bismuth subnitrate may be given during the day. Small doses of ipecac are more than useless; they have been tried in India for over two centuries without lessening the mortality in dysentery. Since more than twenty years the above has been adopted as almost the only treatment in British India and has given the best results.—*Technics.*

**The Treatment of Rheumatism.**—Dr. George L. Peabody treats his cases of acute rheumatism with a combination of salicylic acid and iron, the formula for which was obtained in the following way:

About a year ago a nurse was pouring into a common receptacle some remnants of different medicines, when she noticed that a black precipitate formed by iron was turned into a transparent solution of a rich red hue, as soon as she poured the fluid contents of another bottle. Being a young woman of an inquiring turn of mind, she asked the house physician the cause of this phenomenon. The house staff, to help her in her desire for information, experimented with the drugs that she had been throwing out, and ascertained that her manipulation of the chemicals had been this: She had first poured into the receptacle a salicylic acid. Into this she had poured a solution of iron, with the result of producing a black precipitate. To this she added some sodium phosphate, with the result of producing a clear red solution.

This at once gave a clue to the means of combining iron and salicylic acid without forming a precipitate. The facts were submitted to the apothecary of the hospital, and from them he produced the following formula, which has been in constant use for nearly a year:

℞. Acidi salicylici..............................gr. xx,
Ferri pyrophosphatis..........................gr. v,
Sodii phosphatis..............................gr. j,
Aquæ ..................................℥ ss.

This method of giving this drug in rheumatism has now been fairly tested. It may be said to agree as well with the stomach as any other, and it has the great advantage of not being followed, even if its use be long continued, by the severe anæmia that so often follows the use of salicylic acid, if it be given without iron.

The dose which is described in this formula is given every two hours until improvement justifies a diminunition in the frequency, or until constitutional effects are pronounced.—*Medical News.*

**Hypodermic Injection of Atropine in Hæmoptysis.**—Hasmann advises this method of treatment in desperate cases of hæmoptysis when the use of other remedies has failed. He reports three cases which illustrates this point. One case has suffered from twelve severe hemorrhages in six hours. After the thirteenth the writer injected one-twenty-fifth of a grain of the sulphate of atropine; the hemorrhage did not recur. A similar result was obtained with a lady, in whose case ergotine and preparations of turpentine had failed.

In a third case of persistent hemorrhage through two winters, two injections, of one-twenty-fifth of a grain each, sufficed to check them.—*Reoue Generale de Clinique et de Therapeutique.*

**Aneurism Cured by Position.**—Dr. T. G. Richardson, of New Orleans, at the last meeting of the American Surgical Association, reported the case of a shoemaker, 55 years of age, who was admitted into the hospital for a painful swelling of the left thigh. The tumor, an aneurism of the femoral artery, was about the size of a goose-egg, irregularly flattened. None of the characteristic signs of aneurism were wanting. It was supposed to possess thin walls. The man looked anæmic and delicate. He had had syphilis about nine years before. The cause of the disease was supposed to be the irritation caused by hammering leather on an iron placed on his thigh. The tumor appeared to be inflamed by the manipulation. To overcome this, Dr. Richardson suspended the limb flexed at right angles at the hip and knee. He found on the first day an improvement in the condition of the tumor, and a few days later, that coagulation had occurred. A week later he was discharged, cured, and a few months after only a small nodule could be felt at the site of the tumor. As this is probably the first case of femoral aneurism cured by this method, he desired to call attention to the fact that no pressure was exerted on the tumor, but that the only treatment was flexion, and suspension of the limb, especially the latter. He thought that gravity had a great deal to do with effecting the cure.—*Philadelphia Medical and Surgical Reporter.*

**Abortive Treatment of Whitlow.**—The various methods of treatment recommended as "sure cures" for the painful infliction usually called a felon, are, as a rule, predicated on the physiological fact that *pressure causes absorption;* and, remembering this many years ago, finding that collodion contracts on drying, which it does rapidly by evaporation, I applied it to the end of a finger which presented the symptoms of a felon—the pain and throbbing—and the bowels being constipated, which I have since learned is usually the case, gave a mild cathartic, and realizing that the torture would be increased by the constriction, gave an anodyne sufficient to antagonize this to bridge over the night. The pressure was kept up the second day, when there were a few drops of pus just under the skin, which was liberated, of course without pain, and that was the end of the case.

In an extensive army, hospital and civil practice for twenty-five years this has been the successful method of treating these afflictions: usually without opening. It is well to soften up the parts by strong soap suds, or a weak solution of carbolic acid, before applying the collodion. Poulticing in such cases is highly reprehensible, for if there is any physical wickedness in the system it is attracted to the spot and it hangs on for weeks when it ought to be aborted in a few days.—*Dr. Starnes in Medical Register.*

**Benzoate of Soda in Summer Diarrhœa.**—Dr. A. E. Gregg, of Iowa, speaks highly of the remedy. He says (*in Medical Reporter*): "I will pass by the numerous remedies familiar to us all in the treatment of bowel troubles in children, and speak of the one which I saw recommended by Dr. Wm. B. Watson, of Jersy City, namely, benzoate of soda. During the heated term of last summer I used this remedy in a number of cases of diarrhœa and cholera morbus with most pleasing results. In many cases the vomiting and frequent painful stools ceased within a few hours after giving the first dose. I regret to say that I have not kept a record of the cases in which this treatment was used, and I can therefore give you no detailed account of the results. But I hope the members of this society may deem it worthy of a trial, and that we shall hear more favorably of it in the future.

"The dose is 1 grain for every year of the child's age, up to 15 grains, to be given in water or simple elixir every hour until relief is obtained. I shall not speak of the use of stimulants in these cases, or discuss other methods of treatment, but will conclude by asking my fellow members to give this one remedy a fair test.—*Iowa State Medical Reporter.*

**Neurotic Treatment of Catarrh.**—My plan of treatment for the arrest of catarrh is as follows: I keep a strong solution of bromide (1 in 3) and a bottle of tincture of belladonna (B. P.). When I am conscious of having taken cold, I take 2 to 3 drachms of the bromide solution in a small glass of water—that is to say, 40 to 60 grains of bromide. I repeat this dose in six hours, and, if necessary, take a third dose at a similar interval. Meanwhile, as soon as a flux commences, I take 20 drops (equivalent to 15 minims) of the tincture of belladonna in a little water every hour or two until the throat feels somewhat dry. The painting of the nasal mucous membrane with cocaine solution gives great relief, and powerfully contributes to the cure if the catarrh be severe. Since I hit upon this plan I have never failed rapidly to arrest my own catarrhs, nor have I failed in any instance in which I have myself been able to superintend the administration of the remedies.—*Dr. Lees, in Canada Lancet.*

**Transplantation of Tendon.**—M. Monrod made an observation before the Societe de Chirrurgie, of a case of transplantation of the tendon of a dog to man. The tendon of the medius being severed by a jagged wound, and it being impossible to unite the two ends without causing considerable contraction, a piece of a tendon of a dog was interposed. The result was very good, and the functions of the finger were for the most part re established. M. Trelat said that in a case where it was possible to unite the tendons he pared the edges and brought them together by sutures, and was well satisfied with the result. M. Despres always knew of wounds of tendons to heal by adherence of the two ends to the cicatrix of the skin, and was able to perceive that this method was sufficient to re-establish the functions. In this he was opposed by M. Berger, who maintained that the suture was necessary in certain cases.—*The Medical Press.*

**Ring Pessary in Treatment of Fractured Patella.**—A subscriber writes, "I desire to call attention to a simple, inexpensive and efficient plan of treating fracture of the patella. The only articles required are a gum ring pessary, two strips of adhesive plaster two inches wide and long enough to go round the limb, one above and the other below the knee, and a few yards of muslin bandage.

"Bring the fragments as closely together as possible and encircle with the ring, secure the ring in position by two strips of muslin passing through it and connecting with the adhesive strips. Then bandage well, with this treatment considerable latitude of motion may be allowed the patient in bed without injury, and in most cases he may be allowed to sit up in three weeks.

"Six cases have been treated in this way within the last ten years, all of which have resulted satisfactorily."—*Maryland Medical Journal.*

**Agaricin for Night Sweats.**—Young recommends the following combination:

℞. Agaricini.................................gr. viiij,
Pulv. ipecac et opii........................gr. cxx,
Althæ pulv..................................
Mucilag, acaciæ ........................aa gr. lx,

Met. et div. in pilulæ, No. 100. S. One or two to be taken at night.

In the Bellevue Hospital the following combination has been used with excellent results:

℞. Agaricini (Merck's)............................gr. x,
Atropinæ sulph...............................gr. j,
Acidi sulph. arom...............................*m* mcc.

M. et filter. Dose, 16 minims contain 1-10 of a grain of agaricin, 1-120 of a grain atropine sulphate, and 10 minims of aromatic sulphuric acid. To be given in syrup or simple elixir.—*Kansas City Medical Record.*

**Indications for Thyroidectomy and for Interstitial Injections of Iodine in Parenchymatous Goitre.**—Interstitial injection of iodine is and remains the classical and the French method for the surgical cure of parenchymatous goitre. This treatment is simple, generally efficacious, and almost absolutely innocuous when properly administered. It should be applied to goitres that are rebellious to iodine given internally, and which have become a permanent trouble to the patient or a menace to his existence. It may be applied for æsthetic reasons, should the patient desire it. Thyroidectomy is a redoubtable operation to which recourse should not be had until after repeated and fruitless trials have been made of the intestinal injection of iodine, or in exceptional circumstances, in which the surgeon must be the judge. Only a partial extirpation of the thyroid body is admisible; total extirpation is experimentally condemned.—*Medical Register.*

**Cod-liver Oil in Infantile Atrophy.**—In the *Lancet* Dr. Yeldham, in a letter to the editor, states that in "cases in which no kind of artificial food is of benefit, and the poor little creatures waste away, many of them dying from sheer inanition, cod-liver oil comes in with admirable effect. Let the nurse dip the end of her little finger in the oil, and put it into the child's mouth This may be repeated five or six times in the twenty-four hours. In such small quantities not only does it never disagree, but the child sucks it off the finger with avidity and evident pleasure. It may be administered in this way to the youngest infants. By this simple and inexpensive expedient I have seen many infants, who were absolutely starving for want of the natural food, become plump and happy in an almost incredible short space of time. The oil has the effect of enabling the child to digest other food which it could not take without it. With the aid of cod-liver oil, as I have suggested, any good, pure milk will agree.—*Medical Times.*

**The Prevention of Mammary Abscess.**—Mr. Mial speaks highly of a method preventing mammary abscess. He says that when mammary abscess is on the point of forming, he has frequently seen all the symptoms disappear in a few hours under the influence of fomentations with hot water and carbonate of ammonia. He uses an ounce of the carbonate in a pint of water, and when solution is accomplished, the temperature of the fluid will be hardly too high for fomentation to be commenced with cloths dipped in the liquid. He applies them for from half an hour to two hours, at the same time protecting the nipples. He has often had immediate relief, and seldom requires to make more than three applications.— *Weekly Medical Review.*

**Reduction of Dislocation of the Humerus by Right-angle Traction.**—We notice several reports in the various journals, relative to the ease with which shoulder dislocat on may be reduced by Mr. McLeod's process. It consists in making traction at right angle to the patient's body, steadying the body by the foot, or by any other means the operator chooses. All who have attempted it seem to regard it as highly successful, the reduction being obtained with the minium amount of pain and force. The characteristic "snap" is sometimes wanting.—*Canada Lancet.*

**New Local Anæsthetic.**—Dr. Lervin (*France Medical*) recommends a resinous extract of Kava (piper mephysticum) as a local anæsthetic analogous in its action to cocaine. Applied upon the tongue this extract produces a sensation of heat, quickly followed by anæsthesia. Dropped in the eye it causes slight irritation and an abundant secretion of tears. Complete and lasting anæsthesia of the conjunctiva and of the cornea immediately follows. Hypodermic injections produce insensibility of neighboring tissues without causing any inflammatory phenomena.—*Tech.*

**Stammering.**—Dr. Dio Lewis said: "The worst case of stammering may be cured if the patient be made to mark the time of his speech, as is done in singing. He is at first to beat on every syllable. He begins by reading a piece, striking the finger on the knee at every word. You can also beat time by striking the finger, hitting the thumb against the forefinger, or moving the large toe in the boot. An hour's practice each day will suffice."—*New York Medical Times.*

